# Support for primary care prescribing for adult ADHD in England: national survey

**DOI:** 10.3399/BJGP.2023.0595

**Published:** 2024-09-03

**Authors:** Anna Price, Kieran Becker, John H Ward, Obioha C Ukoumunne, Rebecca Gudka, Anita Salimi, Faraz Mughal, GJ Melendez-Torres, Jane R Smith, Tamsin Newlove-Delgado

**Affiliations:** University of Exeter Medical School, Exeter.; University of Exeter Medical School, Exeter.; University of Exeter Medical School, Exeter, and Department of Psychiatry, University of Oxford, Oxford.; NIHR Applied Research Collaboration South West Peninsula, University of Exeter, Exeter.; University of Exeter Medical School, Exeter.; University of Exeter Medical School, Exeter.; School of Medicine, Keele University, Keele.; University of Exeter Medical School, Exeter.; University of Exeter Medical School, Exeter.; University of Exeter Medical School, Exeter.

**Keywords:** ADHD, general practitioners, prescribing, primary health care, shared care, survey

## Abstract

**Background:**

Attention deficit hyperactivity disorder (ADHD) is a common neurodevelopmental disorder, for which there are effective pharmacological treatments that improve symptoms and reduce complications. Guidelines published by the National Institute for Health and Care Excellence recommend that primary care practitioners prescribe medication for adult ADHD under shared-care agreements with Adult Mental Health Services (AMHS). However, provision remains uneven, with some practitioners reporting a lack of support.

**Aim:**

This study aimed to describe elements of support, and their availability/use, in primary care prescribing for adult ADHD medication in England to improve access for this underserved population and inform service improvement.

**Design and setting:**

Cross-sectional surveys were used to elicit data from commissioners, health professionals (HPs), and people with lived experience of ADHD (LE) across England about elements supporting pharmacological treatment of ADHD in primary care.

**Method:**

Three interlinked cross-sectional surveys were used to ask every integrated care board in England (commissioners), along with convenience samples of HPs and LEs, about prescribing rates, AMHS availability, wait times, and shared-care agreement protocols/policies for the pharmacological treatment of ADHD in primary care. Descriptive analyses, percentages, and confidence intervals were used to summarise responses by stakeholder group. Variations in reported provision and practice were explored and displayed visually using mapping software.

**Results:**

Data from 782 responders (42 commissioners, 331 HPs, 409 LEs) revealed differences in reported provision by stakeholder group, including for prescribing (95% of HPs versus 64% of LEs). In all, >40% of responders reported extended AMHS wait times of ≥2 years. There was some variability by NHS region – for example, London had the lowest reported extended wait time (25%), while East of England had the highest (55%).

**Conclusion:**

Elements supporting appropriate shared-care prescribing of ADHD medication via primary care are not universally available in England. Coordinated approaches are needed to address these gaps.

## Introduction

### Attention deficit hyperactivity disorder and prescribing

Attention deficit hyperactivity disorder (ADHD) is a neurodevelopmental disorder that affects 5–7% of children and adolescents,^1^^,^^2^ and 2–5% of adults.^3^^,^^4^ It is defined by patterns of hyperactivity, impulsivity, and/or inattention that interfere with functioning in daily life, adversely impacting multiple life domains, including physical and mental health, relationships, work, and education.^5^^,^^6^ Higher ADHD prevalence is associated with economic disadvantage and may lead to greater demands on services in some areas.^7^ Despite pharmacological treatments, with substantial evidence of reductions in risks of depression, suicidality, aggression, criminality, and substance-use disorders,^8^^–^12 services for ADHD appear to be insufficient in many regions of the UK. Insufficient services for ADHD are associated with high long-term social, health, personal, and economic costs.^13^ Failure to access pharmacological treatment for those who need it, can have severe impacts on families struggling to manage ADHD, with high emotional and financial costs.^13^ Understanding — and, ultimately, improving — prescribing practices has the potential to benefit the lives of patients and their communities across multiple domains.^14^

**Table:** How this fits in

Improved management of attention deficit hyperactivity disorder (ADHD) in primary care has been proposed as part of the solution to the current ‘failure’ of health care for people with ADHD in England. However, GPs and other primary care professionals need better support from mental health specialists to be able to provide shared-care prescribing of ADHD medication in line with UK guidelines. This research summarises stakeholder-reported availability of the elements necessary for supported and appropriate shared-care prescribing via primary care. Any reorganisation of health care needs to ensure GPs are properly supported to prescribe for patients with ADHD, especially in areas of the country where there are high levels of unmet need.

### Shared-care challenges

The National Institute for Health and Care Excellence (NICE) guidelines for treatment and management of ADHD state that, after titration by a mental health specialist, the prescribing and monitoring of ADHD medication should be carried out under shared-care protocol arrangements with primary care.^15^ Shared care, a formal local agreement between the specialist and the GP, enables GPs to share responsibility for safe prescribing and monitoring of specialist medicines. However, evidence suggests GPs may not feel supported to prescribe under shared care, with challenges including inadequate Adult Mental Health Services (AMHS) provisions and insufficient prescribing guidance.^16^^–^18 GPs may refuse a shared-care agreement if they are not happy with the burden of responsibility and perceived risk.^17^^,^^18^ Perhaps unsurprisingly, patients report multiple challenges in accessing prescriptions for ADHD medication, with access dependent on local service contexts, whether their GP facilitates shared care, and the patient’s ability to advocate for their care.^19^^,^^20^ Recent expert guidance recommends development of ADHD specialisms in primary care to help address these issues.^21^

### Integrating care

The organisation of UK health care, including structural barriers between primary and secondary care, and children’s and adult services, can exacerbate existing health inequalities for people living with ADHD.^13^^,^^22^^–^24 Barriers include difficulties securing a referral for diagnosis, associated waitlists, challenges in transitioning to adult services, difficulties in re-accessing care after dropping out, and variability in whether GPs will prescribe.^13^^,^^24^^,^^25^ The establishment of 42 integrated care systems responsible for jointly planning and delivering health and care services in England — stipulated in the Health and Care Act 2022 — represents an opportunity to improve outcomes and tackle barriers that exacerbate healthcare inequalities for underserved groups, such as people with ADHD. Integrated care boards (ICBs) are now responsible for commissioning primary care services through approximately 1250 primary care networks, each covering populations of 30 000–50 000 patients.^26^ Despite some primary care data evidencing the inconsistent prescribing of ADHD medication,^27^ to date no study has focused on exploring geographic variation in prescribing practice for ADHD, or factors that support successful prescribing.

### Aim

This study aimed to describe the presence of elements that support primary care prescribing of adult ADHD medication across England, incorporating the perspectives of multiple stakeholders.

## Method

### Design

Three linked cross-sectional electronic surveys of primary care provision for people with ADHD were developed following the seven-step method for mapping services^28^ to capture experiences of key stakeholders — namely, commissioners, health professionals (HPs), and people with lived experience of ADHD (LEs). Surveys asked about prescribing, shared care, AMHS, and wait times (Supplementary Box S1). Content was co-designed and iteratively reviewed by the Managing young people (aged 16–25) with Attention deficit hyperactivity disorder in Primary care (MAP) study research advisory groups, to ensure stakeholder priorities were incorporated. Revisions were undertaken to reduce survey completion time (<10 minutes) and ensure accessibility. This study is linked to a wider programme of work by the same authors.^29^

### Participants

Participants comprised:

- commissioners from every ICB in England, who responded in relation to the provision of NHS services in their geographic area;
- HPs working in primary care (for example, GPs, practice managers, and mental health workers), who reported on provision and practice at local levels; and
- LEs (for example, people with ADHD, and their supporters), who responded in relation to personal experience.

Participants had to be >16 years old and living or working in England.

### Recruitment

The commissioner survey was sent via freedom of information (FOI) requests to every ICB in England in October 2022. The anonymous HP and LE surveys (open for 6 weeks from October 2022) were shared via Qualtrics (version 03.23), a secure online survey tool, using a variety of convenience sampling techniques. These included sharing links on the study’s website, via social media (Twitter [now named X] and Facebook), and with research partners (ADHD Foundation and the UK Adult ADHD Network), National Institute for Health and Care Research clinical research networks (CRNs), professional contacts, and other organisations, who distributed them via their networks. Midway through data collection, under-represented regions were identified and subsequent dissemination focused on these areas (by targeting local CRNs and ADHD support groups) with the aim of achieving adequate representation across England.

Data collection and storage were conducted in line with the UK General Data Protection Regulation.

### Data analysis

Data — which comprised responses to questions on prescribing, AMHS availability, wait times, and shared care for people with ADHD from a range of reporting perspectives — were cleaned and analysed in Microsoft Excel and Stata (version 17) by two authors, and checked for accuracy by another. Responders were included in the analysis if they answered the first two survey questions, which meant they provided a postcode, and identified their role. When participants answered ‘do not know’ or missed a response, the data were treated as missing and removed for that outcome. Percentages of responders that reported availability of elements of prescribing practice were presented with 95% confidence intervals (CIs), allowing a comparison of reported provision between stakeholders and by location (ICB or NHS region).

Geographic analyses were carried out using the open-source Quantum Geographic Information System (QGIS) (version 3.26.3) and visual maps were created to display findings. Relevant location data, such as NHS region and longitude/latitude, were obtained from postcodes using a variety of look-up tools and files, such as the *English Indices of Deprivation 2019* (https://imd-by-postcode.opendatacommunities.org/imd/2019), the Office for National Statistics’ Open Geography Portal (https://geoportal.statistics.gov.uk/), and the UK Grid Reference Finder (https://gridreferencefinder.com/postcodeBatchConverter/).

## Results

### Sample

**Figure 1.**
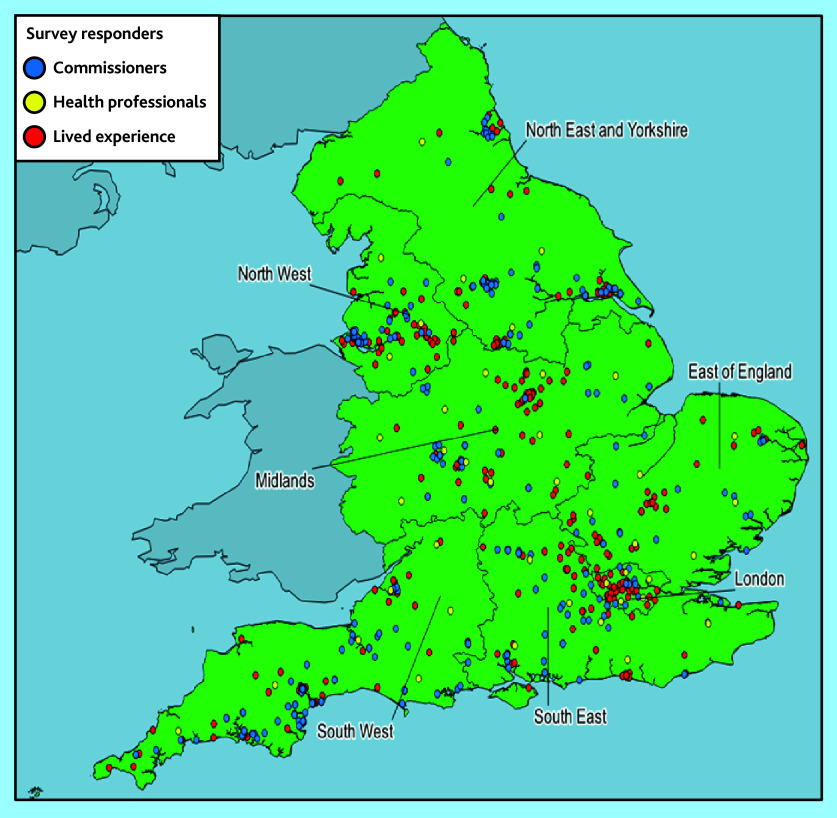
Map showing geographic distribution of survey responders, by NHS region and stakeholder group.

In total, 782 responses were received — these were from commissioners (*n* = 42), HPs working in primary care (*n* = 331), and LEs (*n* = 409) located across England (Table 1 and Figure 1).

**Table 1.** Participants, with sampling method, reporting perspective, and elements of prescribing in primary care on which they reported

**Stakeholder group**	**Sampling method**	**Reporting perspective^a^**	**Elements reported on**	**Responders,** ***n***
Commissioners	Population	Across ICB	AMHS, wait times, shared care	42 (FOI team, *n* = 20; commissioning lead/manager, *n* = 17; programme lead/manager, *n* = 5)^b^
HPs	Convenience	Practice level	Prescribing, AMHS, wait times, shared care	331 (GPs, *n* = 198; nurses, *n* = 51; manager/administration, *n* = 34; other,^c^ *n* = 44)^d^
LEs	Convenience	Personal experience at local practice	Prescribing, AMHS, wait times	409 (LE_P, *n* = 234; LE_S, *n* = 149)^d^

a *Commissioners responded in relation to provision in their ICB (average patient population ∼1.5 million), HPs reported on provision in their practice (average number of patients ∼9400), and LEs reported on individual experiences (population 1).*

b *FOI* res*ponders in commissioning roles focused on general health, mental health, learning disability, and/or autism/neurodevelopment.*

c *’Other’ comprises social prescribers, pharmacists, care coordinators, and health and wellbeing coaches. ADHD = attention deficit and hyperactivity disorder.*

d *Data not provided: HEs,* n *= 4; LEs,* n *= 26. AMHS = Adult Mental Health Services. FOI = freedom of information. HP = health professional. ICB = integrated care board. LE = people with lived experience of ADHD. LE_P = person with ADHD. LE_S = supporter (parents/carers) of person with ADHD.*

### Provision

**Figure 2.**
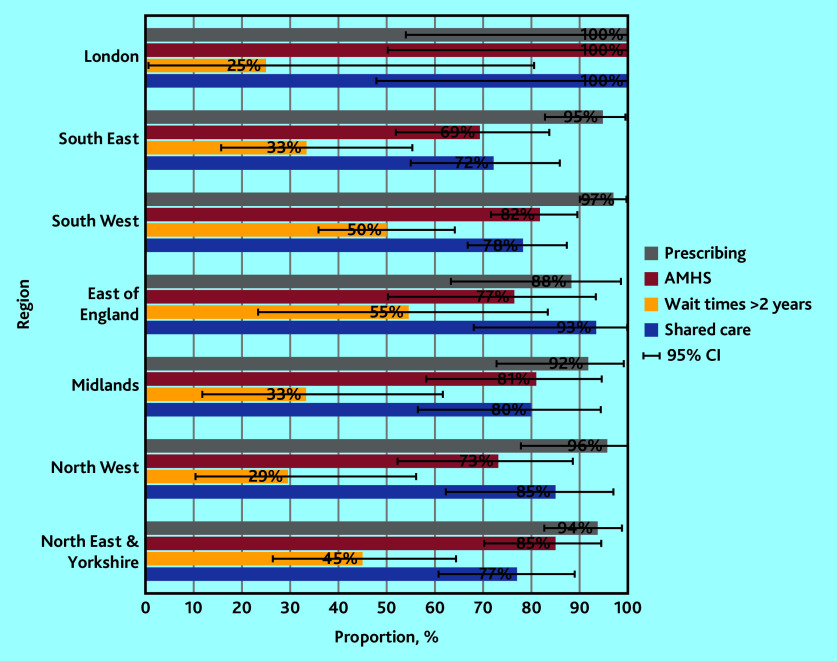
Proportions of health professionals (*n* = 331) reporting elements of support for primary care prescribing of adult ADHD medication by NHS region, with 95% CIs. ADHD = attention deficit and hyperactivity disorder. AMHS = Adult Mental Health Services.

**Figure 3.**
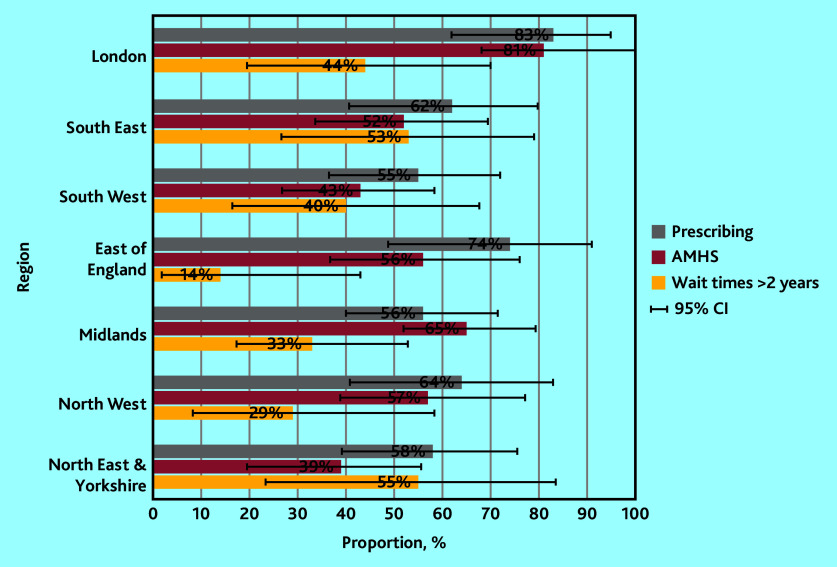
Proportions of patients and supporters with lived experience of ADHD (*n* = 409) reporting ‘elements of support’ for primary care prescribing of adult ADHD medication by NHS region, with 95% CIs. ADHD = attention deficit and hyperactivity disorder. AMHS = Adult Mental Health Services.

Stakeholder responses indicated the availably of elements of support that are necessary and appropriate to support shared prescribing of adult ADHD medication in primary care, with variation by stakeholder group and NHS England (Table 2, Supplementary Table S1, and Figures 2 and 3).

**Table 2.** Reported availability of elements supporting shared-care prescribing of ADHD medication in primary care, by stakeholder group

**Element**	**Stakeholder group**	**Yes, % (nominator/denominator)**	**95% CI**
Prescribing of adult ADHD medication for patients with an NHS diagnosis	HP^a^	89.9 (204/227)	85.2 to 93.5
	LE^b^	38.1 (74/194)	31.3 to 45.4

Prescribing of adult ADHD medication for patients with a private diagnosis	HP	49.2 (90/183)	41.7 to 56.7
	LE	39.5 (68/172)	32.2 to 47.3

AMHS available for patients with ADHD^c^	Commissioners^d^	100 (42/42)	91.6 to 100
	HP	79.2 (179/226)	73.3 to 84.3
	LE	55.5 (132/238)	48.9 to 61.9

Wait lists for AMHS of ≥2 years	Commissioners	45.2 (14/31)	27.3 to 64.0
	HP	42.2 (65/154)	34.3 to 50.4
	LE	37.4 (43/115)	28.6 to 46.9

Shared-care agreement/protocol in place to enable prescribing	Commissioners	90.2 (37/41)	76.9 to 97.3
	HP	79.4 (162/204)	73.2 to 84.7

a *HP working in primary care,* n *= 331.*

b *Patients and their supporter,* n *= 409.*

c *Commissioners and HP reported availability of AMHS for ADHD in their area, LE reported experiences of a referral for ADHD.*

d *Commissioners of primary care,* n *= 42. ADHD = attention deficit hyperactivity disorder. AMHS = Adult Mental Health Services. HP = health professional. LE = person with lived experience of ADHD.*

#### Commissioners

All commissioners reported available AMHS for ADHD in their ICB (Table 2), meaning this was considered available in every NHS region with no geographic variation; however, 45% confirmed extended wait times for these services of ≥2 years (Table 2). Rates of extended wait times varied by NHS region, with the lowest in the Midlands (14%) and London (24%), and the highest in the North West (100%) (see supplementary material). Although 90% of commissioners reported that at least some shared-care protocols were in place, 10% (located in the Midlands, East of England, and the South East), stated that they were not (data not shown).

#### HPs

Prescribing for patients (whether they had an NHS or a private diagnosis) was reported by most HPs, with 95% confirming any prescribing, and little observable difference by NHS region (Figure 2). Interestingly, HPs reported much higher prescribing rates for patients with NHS diagnoses (90%) as opposed to those with private ones (49%) (Table 2). AMHS for patients with ADHD were only reported by 79% of HPs (Table 2), with highest rates in London (100%), and the lowest in the South East (69%) (Figure 2). Extended wait times (≥2 years) were reported by 42% of HPs (Table 2), with the lowest rates in London (25%) and the highest in the East of England (55%) (Figure 2). Similarly, shared-care agreements/protocols were only reported by 79% of HPs (Table 2), with highest rates in London (100%) and the lowest in the South East (72%) (Figure 2).

#### LEs

Any experience of receiving prescriptions for adult ADHD medication (for example, for an NHS and/or for a private diagnosis) was reported by 64% of LEs (data not shown). When explored by reports for an NHS or private diagnosis alone, rates were similar for patients with NHS (38%) or private (40%) diagnoses (Table 2). Proportions of LE responders reporting any prescribing for adult ADHD medication were highest in London (83%), and lowest in the Midlands (56%) and South West (55%) (Figure 3). AMHS referral for ADHD was only confirmed by 56% of LE responders (Table 2), with rates highest in London (81%), and lowest in the South West (43%) and North East and Yorkshire (39%) (Figure 3). Extended wait times were reported by 37% of LEs (Table 2), with the lowest rates in the East of England (14%), and the highest in North East and Yorkshire (55%) and the South East (53%) (Figure 3).

#### Stakeholder variation

**Figure 4.**
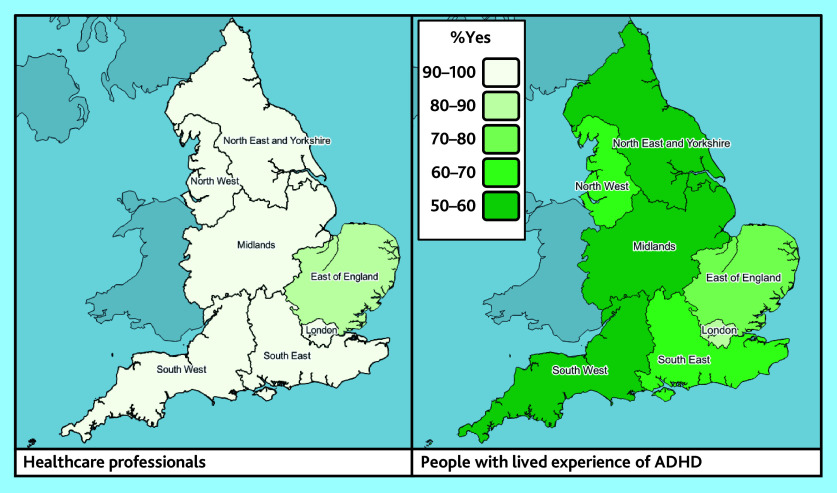
Maps showing proportions of responders reporting the practice of prescribing of adult ADHD medication in primary care, by NHS region and stakeholder group (health professionals, *n* = 331; people with lived experience, *n* = 409). ADHD = attention deficit and hyperactivity disorder. AMHS = Adult Mental Health Services.

There was variation between stakeholder groups in several reported elements of support, with, for example, higher rates of HPs reporting prescribing of adult ADHD medication (95%), compared with LEs (64%) (data not shown). However, there was little observable difference between stakeholder reports of extended wait times for AMHS, which were reported by 45% of commissioners, 42% of HPs, and 37% of LEs respectively (Table 2). Illustrative maps are shown in Figure 4 and Supplementary Figures S1–3.

## Discussion

### Summary

It is perhaps reassuring that 95% of HPs reported that their practices prescribed adult ADHD medication; however, the lower proportions of LEs (64%) that reported any prescribing of ADHD medication, alongside differences between reported prescribing for patients with NHS or private diagnoses, are concerning. Although every commissioner reported having AMHS for patients with ADHD in their ICB, >40% of responders (across stakeholder groups) reported waiting lists of ≥2 years, indicating that timely support for shared-care prescribing is limited.

### Strengths and limitations

This study has numerous strengths, including the use of stakeholder-informed mapping methodology, with survey design guided by stakeholder advisory groups. Use of online self-reported measures was cost-effective and enabled a wide geographic spread of responses. The 100% response rate from ICB commissioners provides an authoritative overview of provision, while the convenience samples of HPs and LEs reflects experiences of key stakeholders (such as GPs, nurses, people with ADHD, and their supporters).

The authors recognise that this study has several limitations. Online data collection may exclude responders with communication difficulties and/or without internet access. In addition, surveys can be subject to recall bias and the convenience sample is likely to have been biased towards responders with high levels of interest in ADHD and/or those experiencing challenges. Further, detailed demographic data were not collected from participants because the authors’ priority was keeping the survey short and engaging for people with ADHD; this may prevent the results from being generalisable to the wider population. Finally, the data only represent a snapshot of provision at the ≥6000 GP practices in England, and relatively low response numbers by region mean that any geographic comparison needs careful interpretation.

### Comparison with existing literature

These data, presented through the lens of primary care, support, extend, and update existing research evidencing the limited availability of AMHS health services for ADHD in the UK,^16^^,^^30^ and lower-than-expected rates of prescribing in children, at transition, and throughout the lifespan.^27^^,^^31^^,^^32^ Reports on the availability of AMHS from ICB commissioners implied that provision of AMHS for ADHD in England may have improved slightly, especially when compared with the fact that just over 90% of clinical commissioning groups (CCGs) reported having AMHS for ADHD in 2018.^19^ However, as ICBs cover a wider geographic area than CCGs, these data are not directly comparable. Variations in reported prescribing provision accord with research using routinely collected data evidencing regional variations in the prescribing of ADHD medication.^27^

### Implications for research and/or practice

This overview of stakeholder perspectives on elements of support for primary care prescribing of ADHD medication provides a useful baseline from which to track the impact of the establishment of ICBs on delivery of the NHS equity agenda.^33^^,^^34^ Regional variations in most elements of support emphasise the importance of the mandated work of newly established ICBs to identify and address unmet need in their areas, especially given the higher rates of ADHD in populations with greater levels of deprivation.^23^^,^^35^ Given that, in 2012, ADHD prevalence for young people was found to be double in areas of greatest deprivation compared with those with least deprivation,^7^ the ICB mandate to tackle inequalities is particularly relevant for this population.^34^

Long wait lists mean that many AMHS are not accessible, regardless of a potential improvement in provision. Where services are limited or unavailable, it is often the patients without family support or resources to enable private health care who are most negatively impacted.^7^^,^^19^ Addressing systems issues, such as AMHS availability and standardisation of shared care nationally, could reduce NHS variation and associated barriers to accessing primary care support for patients with ADHD.

Primary care plays a key role in prescribing and helping to ensure continuity of care for people with ADHD;^19^^,^^20^^,^^36^^–^38 however, GPs need support to feel confident to prescribe.^39^ Even when shared-care protocols are available, GPs may be unwilling to prescribe, because of concerns around robustness of diagnosis or a perception that secondary care monitoring is inconsistent or insufficient.^13^^,^^18^ Qualitative studies have highlighted concerns over the balance of risk and responsibilities in prescribing for ADHD, which were particularly marked where specialist services were lacking.^17^ It is overly simplistic to conclude that GPs need to be better trained, more aware, and more willing to prescribe. One solution to reducing the burden on GPs is providing relevant information in quick, accessible formats that can be integrated seamlessly into existing systems. Information about individual patients’ needs and care pathways should be available at the point of need, in a filtered format, placing minimal demand on GP time and energy. Future research needs to explore solutions from the perspectives of LEs and primary care HPs. Linked qualitative research suggests that digital innovations to support shared care, such as links to shared-care templates and answers to frequently asked questions, might be a cost-effective solution to help primary care providers feel more supported in prescribing for patients with ADHD.^40^

Equitable healthcare provision for adults with ADHD needs to be supported by improving communications across the primary–secondary care interface and providing access to specialist colleagues.^21^^,^^37^ This could be aided by creating standardised shared-care templates, and digital information resources suitable for use by providers and patients.^40^ Future research is planned to gain a better understanding of the barriers to health care for underserved groups.

## Data

Data are stored securely at the University of Exeter and will be made available from the lead author upon reasonable request.
